# Effects of Decomplexation Rates on Ternary Gene Complex Transfection with *α*-Poly(l-Lysine) or *ε*-Poly(l-Lysine) as a Decomplexation Controller in An Easy-To-Transfect Cell or A Hard-To-Transfect Cell

**DOI:** 10.3390/pharmaceutics12060490

**Published:** 2020-05-28

**Authors:** Kyoungnam Kim, Kitae Ryu, Hana Cho, Min Suk Shim, Yong-Yeon Cho, Joo Young Lee, Hye Suk Lee, Han Chang Kang

**Affiliations:** 1 Department of Pharmacy, College of Pharmacy, The Catholic University of Korea, 43 Jibong-ro, Wonmi-gu, Bucheon-si, Gyeonggi-do 14662, Korea; bskn35@naver.com (K.K.); rlxo87@gmail.com (K.R.); hncho0613@gmail.com (H.C.); yongyeon@catholic.ac.kr (Y.-Y.C.); joolee@catholic.ac.kr (J.Y.L.); sianalee@catholic.ac.kr (H.S.L.); 2 Division of Bioengineering, Incheon National University, Incheon 22012, Korea; msshim@inu.ac.kr

**Keywords:** decomplexation, pDNA release, *α*-poly(l-lysine), *ε*-poly(l-lysine), nuclear uptake, transfection difficulty

## Abstract

The tight binding of pDNA with a cationic polymer is the crucial requirement that prevents DNA degradation from undesired DNase attack to safely deliver the pDNA to its target site. However, cationic polymer-mediated strong gene holding limits pDNA dissociation from the gene complex, resulting in a reduction in transfection efficiency. In this study, to control the decomplexation rate of pDNA from the gene complex in a hard-to-transfect cell or an easy-to-transfect cell, either *α*-poly(l-lysine) (APL) or *ε*-poly(l-lysine) (EPL) was incorporated into branched polyethylenimine (bPEI)-based nanocomplexes (NCs). Compared to bPEI/pDNA NCs, the addition of APL or EPL formed smaller bPEI-APL/pDNA NCs with similar zeta potentials or larger bPEI-EPL/pDNA NCs with reduced zeta potentials, respectively, due to the different characteristics of the primary amines in the two poly(l-lysine)s (PLs). Interestingly, although both bPEI-APL/pDNA NCs and bPEI-EPL/pDNA NCs showed similar pDNA compactness to bPEI/pDNA NCs, the addition of APL or EPL resulted in slower or faster pDNA release, respectively, from the bPEI-PL/pDNA NCs than from the bPEI/pDNA NCs. bPEI-EPL/pDNA NCs with a decomplexation enhancer (i.e., EPL) improved the transfection efficiency (TE) in both a hard-to-transfect HepG2 cell and an easy-to-transfect HEK293 cell. However, although a decomplexation inhibitor (i.e., APL) reduced the TE of bPEI-APL/pDNA NCs in both cells, the degree of reduction in the TE could be compensated by PL-mediated enhanced nuclear delivery, particularly in HepG2 cells but not HEK293 cells, because both PLs facilitate nuclear localization of the gene complex per its cellular uptake. In conclusion, a decomplexation rate controller could be a potential factor to establish a high TE and design clinically available gene complex systems.

## 1. Introduction

As the significance of the application of genetic material for treating various disorders has increased, nanoscale nonviral gene complexes have been extensively and intensively investigated for the last three decades by using newly synthesized or naturally found gene carriers and by introducing functional moieties [1,2,3,4]. Although a lipid-based siRNA delivery system (i.e., Onpattro) was approved by the US FDA in August, 2018, most gene delivery systems still have not been able to reach optimal or acceptable transfection levels for clinical applications [1,2]. One compelling reason is that nanosized gene complexes go through multiple individual rate-limiting steps during the delivery of genetic materials in cells and animals. The complexes (particularly, hydrophobic or cationic complexes) can bind with plasma proteins (e.g., albumin and opsonins) or can be scavenged by phagocytic cells in blood. Particularly, these rate-limiting steps consecutively occur at the cellular and intracellular levels and are comprised of noncontact or contact cellular interactions, cellular internalization (e.g., endocytosis, membrane penetration), endosomal/endolysosomal/lysosomal escape, intracellular trafficking, nuclear or mitochondrial translocation (depending on the subcellular gene delivery target), and unpacking of gene complexes and finally gene release from the complexes [2,4,5]. Among these various obstacles, both a decomplexation step between the gene and gene-holding material and a gene-releasing step from the gene complex are particularly required at the correct location and time in the cellular transfection process [5,6,7,8].

To select the correct place for gene release, four important limitations have been considered: (1) nuclease-mediated degradation, (2) poor cellular internalization, (3) almost stationary cytosolic transport, and (4) limited targeted organelle entry of genes (particularly nuclear entry) [9]. First, nucleases in the blood and cytosol could hydrolyze a naked gene and cause further fragmented genes to lose their transcriptional information [10,11]. Second, negatively charged genes can rarely cross negatively charged plasma membranes due to electrostatic repulsion [12]. Third, a naked gene in the cytosol could rarely diffuse and approach a neighboring nucleus [13] because the electrostatic repulsion between the negative charges in the gene causes the gene to be expanded up to the micron scale [12,14] and because both cytosolic crowding and viscosity strongly limit cytosolic transport of the gene [13]. Fourth, if a gene does not have a nuclear targeting moiety or if the nuclear membrane is not temporarily broken during mitosis, the expanded gene is not small enough to pass through the nuclear pores in the nuclear membrane, leading to poor nuclear entry [15]. Of course, the four addressed issues are not required to be resolved for all types of genes because the correct place for the pDNA is the nucleus, and for the siRNA and miRNA, it is the cytosol. In particular, pDNA is affected by all the issues, the two RNAs are influenced only by the first two issues. Thus, efficient pDNA delivery systems should protect the gene before reaching the nucleus but should release the gene in the nucleus, because the gene carrier should be able to avoid the successive issues that a naked gene encounters in the extracellular, cellular, and intracellular compartments.

To selectively release genes at the right place (e.g., the nucleus for most pDNA), a balance between the holding and releasing of genes in gene complexes has to be controlled by either endogenous or complex components. First, endogenous components include natural macromolecules (e.g., DNA, RNA, proteins) and intracellular chemicals/ions/radicals (e.g., pH, enzymes, glutathione (GSH), reactive oxygen species (ROS)). Negatively charged endogenous macromolecules could competitively detach positively charged carriers from gene complexes to act as a gene release mechanism [16] because most gene complexes have positive zeta potentials. Intracellular pH values in acidic (e.g., endosomes, lysosomes) and neutral (e.g., cytosol, nucleus) environments could neutralize the negative charges (e.g., phosphate) of the genes and the positive charges (e.g., amine, imine) of the carrier polymers, respectively, reducing gene-holding interactions. Additionally, intracellular stimuli (e.g., pH, enzymes, GSH, ROS) could fragmentize carrier polymers to decrease gene complexation because longer carrier polymers generally possess stronger gene-holding forces than shorter fragments [17]. Second, in complex components (e.g., genes, carrier polymers), tuning carrier polymers have been considered due to limited modification of the genes. As mentioned above, introducing positively charged moieties [18,19] and intracellular stimuli-cleavable chemical linkers [17,20,21] into carrier polymers could affect gene holding or releasing interactions. Additionally, certain hydrophobic moieties within carrier polymers could be introduced to control gene-holding forces in gene complexes [22].

In general, a shorter charged macromolecule forms a looser complex with a long countercharged macromolecule than the complex formed with a longer charged macromolecule [8,23]. Based on this fact, additional charged macromolecules could be introduced into two-component gene complexes to construct gene complexes with three or more components. As a result, this multicomponent gene complex could have strong gene-holding or gene-releasing characteristics depending on additional components. For example, when mixing siRNA and a long *α*-poly(l-lysine) (APL), the addition of pDNA formed a tighter APL/pDNA-siRNA complex than the APL/siRNA complex [23]. Similarly, when pDNA was complexed with a mixture of a long APL and a long reducible APL (RAPL; synthesized by the oxidation of Cys-(Lys)_10_-Cys), the APL-RAPL/pDNA complex represented quicker pDNA release than that of the APL/pDNA complexes because RAPL was fragmented into the short Cys-(Lys)_10_-Cys sequence in the cytosol and nucleus, and the fragments had relatively weaker gene-holding activity than that of RAPL [8]. In particular, our recent studies have reported different complexing/decomplexing abilities of two lysine-based polymers (i.e., APL and *ε*-poly(l-lysine) (EPL)) [7,24]. Although two poly(l-lysine) (PL) polymers have the same repeating unit (i.e., l-lysine), the primary amines at the *α*- and *ε*-positions of _L_-lysine participate in the formation of amide bonds, and two resultant PLs have gene-complexable primary amines with different pK_a_ values: 9–10 for APL and 7.6 for EPL [24,25]. The different pK_a_ values (i.e., different positive charges at neutral pH values) create a tighter complexation and slower decomplexation for the APL/pDNA complex, unlike the looser complexation and faster decomplexation of the EPL/pDNA complex [24]. Interestingly, when applying both APL and EPL to construct the APL-EPL/pDNA complex, its complexation and decomplexation were strongly influenced by the amount of APL or EPL in the ternary complex [7]. Understanding the effects of decomplexation rates on nonviral transfection efficiency has mostly focused on tuning carrier components.

Although the same nonviral gene complexes have been used in cells, their different transfection efficiencies have been reported depending on the cell type [26,27,28]. This fact indicates that the gene release rates of gene complexes could differentially affect cells to express transgenes depending on the cell. HEK293 cells, a human embryonic kidney cell line, have often been used to transfect genes of interest in biological experiments and to produce recombinant proteins due to their high and stable gene expressing capability [29,30]. On the other hand, although HepG2 cells (a human hepatocellular carcinoma cell line) have been used as a well-known and suitable in vitro model of human hepatocytes to understand cellular functions and disease mechanisms [31,32], their gene of interest expression levels are poor. Thus, in this study, when transfecting easy to transfect cells (i.e., HEK293 cells) or hard to transfect cells (i.e., HepG2 cells), the effects of decomplexation on transfection efficiency were investigated. To avoid endosomal sequestration of the gene complex and to maximize the difference between gene-holding and gene-releasing capability, either APL as a slow gene-releasing (i.e., strong gene-holding) carrier or EPL as a fast gene-releasing (i.e., weak gene-holding) carrier was incorporated into branched polyethylenimine (bPEI; the gold standard polymeric transfection reagent)-based gene complexes. After the resultant ternary bPEI-APL/pDNA and bPEI-EPL/pDNA complexes were constructed, their physicochemical characteristics (e.g., size, zeta potentials, gene condensation) and biological characteristics (e.g., cellular uptake, nuclear uptake, transfection efficiency) in HEK293 or HepG2 cells were investigated.

## 2. Materials and Methods

### 2.1. Materials and Cell Culture

Two poly(l-lysine) (PL) polymers were purchased: *α*-poly(l-lysine) hydrobromide (APL·HBr; molecular weight (MW) 4–15 kDa as measured by viscosity) was purchased from Sigma-Aldrich (St. Louis, MO, USA) and *ε*-poly(l-lysine) hydrochloride (EPL·HCl; MW 3.5–4.7 kDa measured by MALDI-TOF) was purchased from Zhengzhou Bainafo Bioengineering Company (Henan, China). Branched polyethylenimine (bPEI_25kDa_; Mw 25 kDa, M_n_ 10 kDa), heparin sodium salt, 4-(2-hydroxy-ethyl)-1-piperazine (HEPES), d-glucose, sodium bicarbonate, Dulbecco’s modified Eagle’s medium (DMEM), Ca^2+^-free and Mg^2+^-free Dulbecco’s phosphate-buffered saline (DPBS), fetal bovine serum (FBS), penicillin-streptomycin antibiotics, trypsin-EDTA solution, Hoechst 33342, formalin, and the Nuclei PURE Prep nuclei isolation kit were purchased from Sigma-Aldrich (St. Louis, MO, USA). For gene staining, two intercalating dyes, ethidium bromide (EtBr) and YOYO-1, were purchased from Sigma-Aldrich (St. Louis, MO, USA) and Invitrogen, Inc. (Carlsbad, CA, USA), respectively. Plasmid DNA (pDNA) encoding firefly luciferase (gWiz-Luc or pLuc) was purchased from Aldevron, Inc. (Fargo, ND, USA). The Pierce^TM^ BCA protein assay kit and luciferase assay kit were obtained from Thermo Fisher Scientific (Waltham, MA, USA) and Promega Corporation, Inc. (Madison, WI, USA), respectively.

HEK293 cells and HepG2 cells were purchased from Korean Cell Line Bank (Seoul, Korea). Using complete culture medium, the two cell lines were cultured at 37 °C in humidified air containing 5% CO_2_. The culture medium was prepared by adding d-glucose (4.5 g/L), 10% FBS, and 1% antibiotics in DMEM.

### 2.2. Preparation of the Nanocomplex

To generate two ternary bPEI-PL/pDNA nanocomplexes (NCs) (i.e., bPEI-APL/pDNA NC and bPEI-EPL/pDNA NC), a cationic solution containing both bPEI and PL (i.e., APL or EPL) and an anionic pDNA solution ([pDNA] = 0.1 μg/μL) in HEPES buffer (20 mM, pH 7.4) were prepared separately to an equal volume (10 μL). After the two charged solutions were mixed, the solution was vortexed for 15 s and then incubated for 30 min at room temperature (RT; 23 ± 2 °C) to generate the NC solutions (20 μL) consisting of either bPEI-APL/pDNA NC or bPEI-EPL/pDNA NC. As a control binary NC, bPEI/pDNA NC was prepared by mixing a cationic bPEI solution with an anionic pDNA solution. In this study, the complexation ratio of all NCs was prepared at a fixed N/P ratio of 5, using the amine (N) group of the polycations (i.e., bPEI, APL, or EPL) and the phosphate (P) group of pDNA. For a ternary bPEI-PL/pDNA NC, if bPEI-PL(20%)/pDNA NC was expressed, 80% and 20% of the amine (N) groups were from bPEI and the PL (APL or EPL), respectively. The resultant NC solutions ([pDNA] = 0.05 μg/μL) with an N/P of 5 were used for further studies.

### 2.3. Particle Size and Zeta Potential of Nanocomplexes

Each NC solution (100 μL; 5 μg pDNA) was diluted with HEPES buffer (20 mM, pH 7.4) to adjust the concentration to 5 μg/mL pDNA in the NC solution (1 mL). The NCs in solution were monitored by using a zeta potential and particle size analyzer (ELS-Z; Photal Otsuka Electronics Co., Osaka, Japan) at a wavelength of 677 nm and a constant angle of 90° at RT. The particle size was estimated based on the refractive index (1.33) for all aqueous samples. These data are expressed as a number-average particle sizes.

In addition, 3 μL of an NC-containing solution ([DNA] = 0.1 mg/mL) was dropped on a grid and dried at RT for 12 h. Then, the dried NCs were imaged by a transmission electron microscope (TEM; JEM1010; JEOL, Tokyo, Japan).

### 2.4. Dye Quenching-Based pDNA Compactness Assay

The compactness of pDNA in the NCs was evaluated by the fluorescent EtBr dye intercalating into pDNA being quenched by polycation-mediated NC formation. After one EtBr molecule per 10 pDNA bases was added into the pDNA solution, EtBr-intercalated pDNA was prepared. A ternary bPEI-PL/pDNA NC was constructed by using EtBr-intercalated pDNA, and the NC was incubated at RT for 4 h. The compactness of pDNA in each NC was evaluated by measuring the fluorescence intensity (F) of EtBr at 510 nm (excitation) and 595 nm (emission) and calculated by the following equation.

$$\mathrm{Compactness}\;\mathrm{of}\;\mathrm{pDNA}\;\mathrm{in}\;\mathrm{NC}\;\left(\%\right)=\left(1-\frac{F_{\mathrm{NC}}-F_{\mathrm{buffer}}}{F_{\mathrm{EtBr}-\mathrm{pDNA}}-F_{\mathrm{buffer}}}\right)\times 100$$

Here, F_NC_, F_EtBr-pDNA_, and F_buffer_ indicate the fluorescence intensities of EtBr-pDNA in the NC, EtBr-pDNA in buffer, and buffer, respectively.

### 2.5. Heparin-Induced Decomplexation of the Nanocomplexes

After the NC was decomplexed by negatively charged heparin, its pDNA release was monitored by a gel electrophoresis assay. The NC was added to a decomplexation solution that contained heparin sodium salt (0–100 μg/mL) in 150 mM aqueous NaCl and then incubated at 37 °C for 30 min. After the decomplexed NC solution (10 μL; [pDNA] = 12.5 μg/mL) was loaded into an 0.8% agarose gel with EtBr, the NC-loaded gel in 0.5 × TBE buffer was run at 100 V for 1 h. Finally, decomplexation of the NC was evaluated by a UV illuminator. Particularly, to evaluate the exposure and release of pDNA from the NC following heparin challenge, the fluorescence blot of the decomplexed pDNA was compared with that in which no pDNA was detected (0%) and in which all the loaded pDNA was detected (100%) by a densitometry method. The amount of pDNA detected in the loading well and that of the electrophoresed pDNA were regarded as the exposed pDNA and the released pDNA from the NC, respectively.

### 2.6. Transfection Efficiency and Cytotoxicity of the Nanocomplex

HEK293 cells or HepG2 cells were seeded at a cell density of 1 × 10^5^ or 5 × 10^5^ cells/well, respectively, in a 6-well plate. After the seeded cells were incubated in complete culture medium for 24 h, the complete medium (2 mL) was replaced with serum-free culture medium (i.e., transfection medium) for 1 h before NC treatment. Then, the NC solution (20 μL; [pDNA] = 0.05 μg/μL) was added to the cells, and the NC-transfected cells were incubated for 4 h in transfection medium. After the serum-free medium was replaced with serum-supplemented complete culture medium, the cells were incubated for an additional 44 h. Forty-eight hours post-transfection, the NC-transfected cells were rinsed twice with Ca^2+^-free and Mg^2+^-free DPBS and then lysed with reporter lysis buffer. After following the protocols of the Pierce^TM^ BCA protein assay kit and luciferase assay kit, the protein contents and the relative luminescent unit (RLU) of the NC-transfected cells were evaluated. The transfection efficiency (TE) and normalized TE (NTE) of bPEI-PL/pDNA NC were compared to those of the control NC (bPEI/pDNA NC) and estimated using the following equations.

$$\mathrm{TE}\;\mathrm{of}\;\mathrm{NC}=\frac{\mathrm{RLU}\;\mathrm{of}\;\mathrm{NC}\text{-}\mathrm{transfected}\;\mathrm{cells}}{\mathrm{protein}\;(\mathrm{mg})\;\mathrm{of}\;\mathrm{NC}\text{-}\mathrm{transfected}\;\mathrm{cells}}$$

$$\mathrm{NTE}\;\mathrm{of}\;\mathrm{sample}\;\mathrm{NC}=\frac{\mathrm{TE}\;\mathrm{of}\;\mathrm{sample}\;\mathrm{NC}}{\mathrm{TE}\;\mathrm{of}\;\mathrm{control}\;\mathrm{NC}}$$

For the in vitro cytotoxicity study, HEK293 cells or HepG2 cells were seeded at a cell density of 1.25 × 10^4^ or 6.25 × 10^4^ cells/well, respectively, in a 48-well plate, and the concentration of pDNA in the NCs was 0.125 μg in 0.25 mL. After transfecting the cells with NCs, cytotoxicity experiments were performed similarly to the in vitro transfection experiments. Upon completing 48 h-transfection procedures, MTT solution (25 μL of 5 mg/mL) was added to the medium, and the cells were incubated for an additional 4 h. After discarding the medium, the living cell-producing formazan crystal was dissolved in DMSO (0.25 mL), and their absorbance was monitored at 570 nm. The viability of NC-transfected cells was calculated by the following equation:

$$\mathrm{Cell}\;\mathrm{viability}\;(\%)\;\mathrm{of}\;\mathrm{NC}=\frac{\left(\mathrm{Absorbance}\;\mathrm{of}\;\mathrm{NC}\text{-}\mathrm{transfected}\;\mathrm{cells})-(\mathrm{Absorbance}\;\mathrm{of}\;\mathrm{DMSO}\right)}{\left(\mathrm{Absorbance}\;\mathrm{of}\;\mathrm{untransfected}\;\mathrm{cells})-(\mathrm{Absorbance}\;\mathrm{of}\;\mathrm{DMSO}\right)}\times 100\;\left(\%\right)$$

### 2.7. Proton Buffering Capacity of the Mixtures of bPEI and PL

To estimate the proton buffering capacity of the mixture of bPEI and PL in bPEI-PL/pDNA NCs, the mixture was dissolved in 150 mM NaCl. The mixed polymer solution was adjusted to pH 9 by adding 1 N NaOH (aq). After the addition of 0.1 N HCl (aq), the pH change of the mixed polymer solution was monitored. In the endosomal pH range (i.e., pH 5.1–7.4), its proton buffering capacity was calculated by the following equation:

$$\mathrm{proton}\;\mathrm{buffering}\;\mathrm{capacity}=\;\frac{\Delta V_{0.1N\;\mathrm{HCl}}\times C_{\mathrm{HCl}}}{m_{\mathrm{polymer}}}$$

In the equation, $\Delta V_{0.1N\;\mathrm{HCl}}$ is the volume of 0.1 N HCl (aq) required to decrease the pH of the mixed polymer solution from 7.4 to 5.1; $C_{\mathrm{HCl}}$ is the concentration of HCl (aq); and $m_{\mathrm{polymer}}$ is the moles of the polymer mixture in the mixed polymer solution.

### 2.8. Cellular Uptake and Nuclear Uptake of the Nanocomplexes

HEK293 cells or HepG2 cells were seeded at a cell density of 1 × 10^5^ or 5 × 10^5^ cells/well, respectively, in a 6-well plate and then incubated in complete culture medium (2 mL) for 24 h. One hour before NC transfection, the complete medium was replaced with serum-free transfection medium. After a ternary bPEI-PL/pDNA NC was prepared by using YOYO-1-intercalated pDNA, the NC solution (20 μL; [pDNA] = 0.05 μg/μL) was added to the cells. After 4 h of incubation, the NC-transfected cells were rinsed twice with Ca^2+^-free and Mg^2+^-free DPBS and then used for either cellular uptake (CU) or nuclear uptake (NU) studies.

For CU, the rinsed cells were detached by trypsin-EDTA solution, pelletized by centrifugation, resuspended in DPBS (300 μL), and then fixed with 4% formalin solution. For NU, nuclei were isolated from NC-transfected cells by using the protocols of the Nuclei PURE Prep nuclei isolation kit. Briefly, an ice-cold lysis solution was added to the rinsed cells, and the cells were collected with a cell scraper and transferred to a 1.5 mL tube. After sucrose cushion solution (1.8 M) was added to the harvested cell solution in the tube, the tube was centrifuged at 4 °C and 13,000 rpm for 45 min. The pelletized nuclei were resuspended in DPBS (300 μL) and fixed with 4% formalin solution. The YOYO-1 fluorescence in the NC-transfected cells or nuclei was measured by a flow cytometer (FACSCanto II, Becton-Dickinson, Franklin Lakes, NJ, USA) equipped with a primary argon laser (488 nm) and a fluorescence detector (530 ± 15 nm).

In this study, bPEI/pDNA NC (N/P 5) was used as the control NC. The normalized CU (NCU), normalized NU (NNU), and nuclear preference with respect to CU (NP/CU) of the bPEI-PL/pDNA NC were estimated using the following equations.

$$\mathrm{NCU}\;\mathrm{of}\;\mathrm{sample}\;\mathrm{NC}=\frac{\mathrm{CU}\;\mathrm{of}\;\mathrm{sample}\;\mathrm{NC}}{\mathrm{CU}\;\mathrm{of}\;\mathrm{control}\;\mathrm{NC}}$$

$$\mathrm{NNU}\;\mathrm{of}\;\mathrm{sample}\;\mathrm{NC}=\frac{\mathrm{NU}\;\mathrm{of}\;\mathrm{sample}\;\mathrm{NC}}{\mathrm{NU}\;\mathrm{of}\;\mathrm{control}\;\mathrm{NC}}$$

$$\mathrm{NP}/\mathrm{CU}\;\mathrm{of}\;\mathrm{sample}\;\mathrm{NC}=\frac{\mathrm{NNU}\;\mathrm{of}\;\mathrm{sample}\;\mathrm{NC}}{\mathrm{NCU}\;\mathrm{of}\;\mathrm{sample}\;\mathrm{NC}}$$

### 2.9. Intracellular Localization of the Nanocomplexes

HEK293 cells or HepG2 cells were seeded at a cell density of 6 × 10^3^ or 3 × 10^4^ cells/well, respectively, in a covered glass bottom dish and then incubated in complete culture medium for 24 h. One hour before polymeric transfection, the culture medium was replaced with serum-free transfection medium. After a ternary bPEI-PL/pDNA NC was prepared by using YOYO-1-intercalated pDNA, the NC solution (0.2 μg of pDNA in 20 μL) was added to the cells. After 4 h of incubation, the polyplex-transfected cells were rinsed twice with Ca^2+^-free and Mg^2+^-free DPBS. Additionally, 10 min prior to the end of the 4 h incubation, Hoechst 33342 (0.2 μg/mL) for nuclear staining was added to the medium. The rinsed cells were monitored by a laser scanning confocal microscope equipped with excitation lasers (408 nm for the diode and 488 nm for Ar) and variable bandpass emission filters (LSM710, ZEISS, Oberkochen, Germany). The nuclear localization of YOYO-1-stained pDNA delivered by the NC was quantified by the fluorescence of two regions of interest (ROIs) (i.e., the cell and the Hoechst 33342-stained nucleus), and their fluorescence intensities were calculated by using ImageJ software. The fluorescence baseline was determined from the autofluorescence of untreated cells. The nuclear localization of YOYO-1-stained pDNA was calculated by the following equation.

$$\mathrm{Nuclear}\;\mathrm{localization}\;\mathrm{of}\;\mathrm{pDNA}\;\left(\%\right)=\frac{\mathrm{YOYO}\text{-}1\;\mathrm{labeled}\;\mathrm{pDNA}\;\mathrm{pixels}\;\mathrm{in}\;\mathrm{nucleus}}{\mathrm{YOYO}\text{-}1\;\mathrm{labeled}\;\mathrm{pDNA}\;\mathrm{pixels}\;\mathrm{in}\;\mathrm{cell}}\times 100$$

### 2.10. Statistical Analysis

For statistical analysis, the data were evaluated by an unpaired Student’s *t*-test. The significance is expressed at a confidence level of *p* < 0.05.

## 3. Results and Discussion

### 3.1. Decomplexation of the bPEI-PL/pDNA Nanocomplex

**Figure 1:**
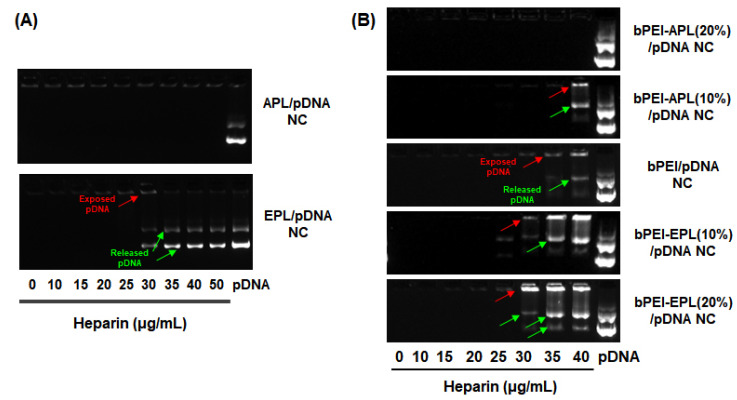
Heparin-induced decomplexation of (**A**) poly(l-lysine) (PL)/pDNA nanocomplexes (NCs) and (**B**) branched polyethylenimine (bPEI)-PL/pDNA NCs. After the polyplexes were exposed to heparin-containing aqueous NaCl (150 mM) at 37 °C for 30 min, the polyplexes were electrophoresed in 0.8% agarose gel. Red- or green-colored arrows indicate exposed or released pDNA, respectively.

Prior to the evaluation of decomplexation of the bPEI-PL/pDNA NCs, the different decomplexation rates of the PL-based NCs were reconfirmed because the experimental conditions (e.g., incubation time) in this study were different from those in a previous study [24]. As shown in Figure 1A, after preparing APL/pDNA NC and EPL/pDNA NC at a fixed N/P ratio of 5, the NCs were exposed to heparin in 150 mM aqueous NaCl at 37 °C for 30 min. When heparin was not present, the two PL/pDNA NCs completely shielded the pDNA because exposed or uncomplexed pDNA was not detected by EtBr-mediated fluorescence. However, when increasing the concentration of heparin from 0 to 50 μg/mL, the two PL/pDNA NCs showed different decomplexation or pDNA release patterns. APL/pDNA NC did not show any decomplexed (exposed and released) pDNA from the NC at 50 μg/mL heparin, whereas EPL/pDNA NC partially released pDNA (approximately 40%) at 30 μg/mL heparin and substantial pDNA (>75%) was released at heparin concentrations greater than 35 μg/mL. These results indicate that APL has a strong electrostatic attraction to pDNA, unlike the weak pDNA electrostatic attraction of EPL, which is in agreement with previous results [24]. Thus, APL and EPL could be used as a decomplexation inhibitor and a decomplexation enhancer, respectively.

When the two decomplexation controllers (APL and EPL) were added into the bPEI-based NCs, their different decomplexation rates were monitored by heparin-induced decomplexation studies (Figure 1B). In general, heparin-induced decomplexation causes either the exposure of pDNA on the surface of the NC or the release of pDNA from the NC. When heparin was applied to bPEI/pDNA NCs (N/P 5) as a control polyplex, 35 μg/mL heparin caused approximately 9% of the pDNA to be exposed on the surface of the NC. In the presence of 40 μg/mL heparin, approximately 17% and 8% pDNA was exposed on the surface of the NC and released from the NC, respectively. Moreover, when bPEI-APL/pDNA NC was exposed to heparin, the decomplexed pDNA decreased with increasing APL content in the NC. bPEI-APL(10%)/pDNA NC showed approximately 35% pDNA decomplexation (16% exposed and 19% released) after treatment with 40 μg/mL heparin, whereas heparin did not induce decomplexation of pDNA from the bPEI-APL(20%)/pDNA NC. On the other hand, the bPEI-EPL/pDNA NC exposed to heparin resulted in more decomplexation than the bPEI/pDNA NC. Decomplexation of bPEI-EPL(10%)/pDNA NC occurred by exposure (approximately 9%) of pDNA at 30 μg/mL heparin and both exposure (approximately 42% or more) and release (approximately 23% or more) of pDNA at heparin concentrations of 35 μg/mL or more. Higher EPL contents in bPEI-EPL/pDNA NC reduced the required amount of heparin for exposure and release of pDNA from the NC. These results suggest that the presence of APL or EPL in bPEI-based NCs results in less or more decomplexation of pDNA, respectively, than the decomplexation of bPEI/pDNA NC. This phenomenon could be caused by the different characteristics (e.g., pK_a_, positive charges) of the primary amines in the two PLs investigated in our previous studies [7,24].

### 3.2. Physicochemical Characteristics of the bPEI-PL/pDNA Nanocomplexes

Although different decomplexation rates of bPEI-PL/pDNA NCs were exhibited in intracellular compartments (particularly the nucleus), NCs should completely protect pDNA until it reaches the nucleus. To pursue this aim, the NCs should have compact binding pDNA that is small in size.

**Figure 2:**
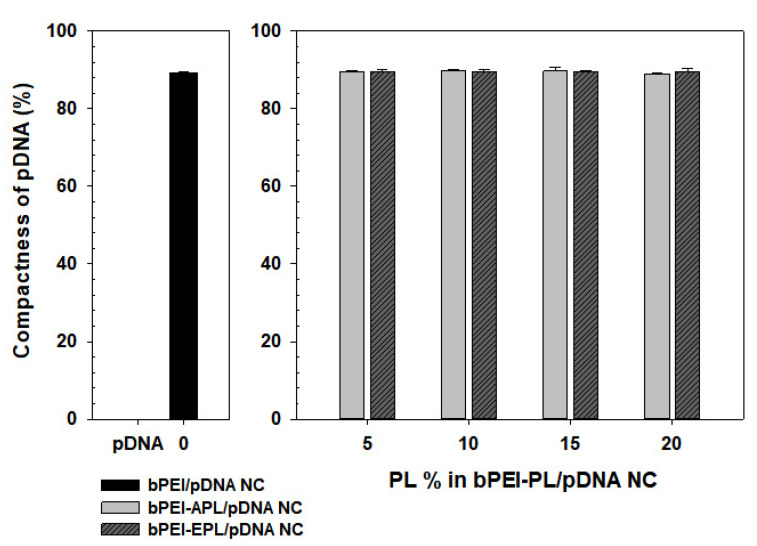
Compactness of pDNA in bPEI-PL/pDNA NCs. The data are expressed as the mean ± standard deviation (SD) (*n* = 3).

First, the compactness of pDNA in each NC was evaluated by polycation-mediated quenching of EtBr intercalated pDNA (Figure 2). To estimate the compactness of the pDNA in the NC(%), the free pDNA intercalated with EtBr was set as 0% because its EtBr fluorescence intensity was the highest, and none of the fluorescence measures could theoretically be 100% because polycations can completely compact the pDNA to the strongest possible complexation level. Based on this assumption, the estimated compactness of the pDNA in the bPEI/pDNA NC (%) was approximately 89%. Interestingly, the addition of APL or EPL (with a limited amount of 0–20%) into the bPEI-based NCs did not improve or reduce pDNA compactness. Regardless of the PL content (0–20%), both the bPEI-APL/pDNA NC and bPEI-EPL/pDNA NC had approximately 89–90% pDNA compactness.

**Figure 3:**
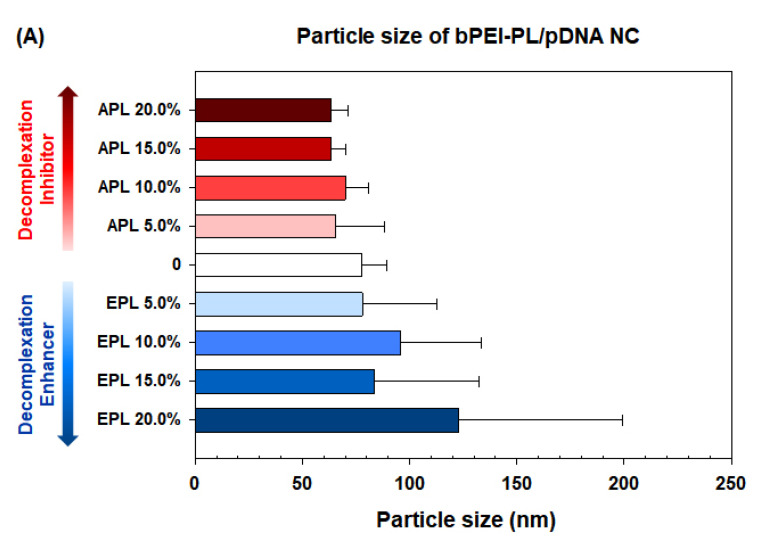

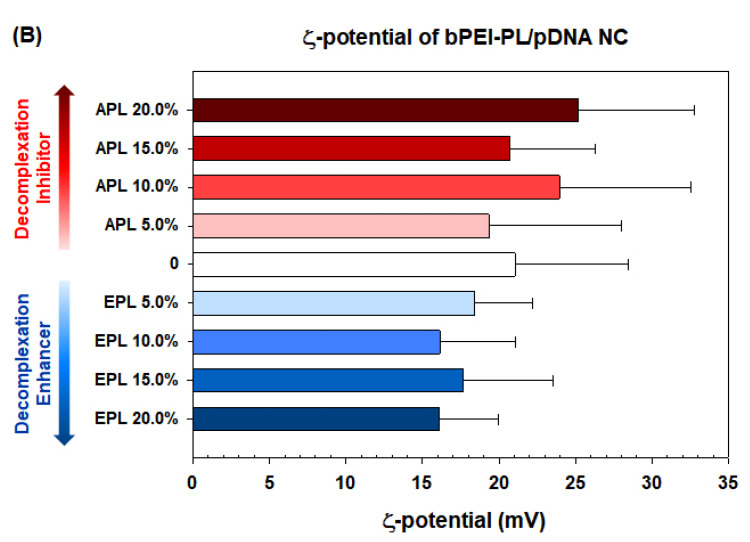
(**A**) Particle size and (**B**) zeta potential of bPEI-PL/pDNA NCs. The data are expressed as the mean ± SD (*n* = 3).

Second, the control polyplex, bPEI/pDNA NC (N/P 5), had a number-average diameter of approximately 78 nm, as measured by a light scattering (LS) technique. As expected, the addition of APL formed a smaller NC, whereas EPL formed a relatively larger NC (Figure 3A). The particle sizes of bPEI-APL(5–20%)/pDNA NCs were 80–90% the size of the bPEI/pDNA NC regardless of the APL content, and had a unimodal distribution with a polydispersity of between 0.17 and 0.25 (Figure S1). However, with increasing EPL content in bPEI-EPL/pDNA NCs, the sizes of the NCs increased: approximately 78 nm for the bPEI-EPL(5%)/pDNA NC and approximately 123 nm for the bPEI-EPL(20%)/pDNA NC. Additionally, the TEM images showed that the dried bPEI-PL/pDNA NCs were slightly smaller, by 20–30%, than the hydrated NCs (as determined by LS) and were spherical (Figure S2). Nevertheless, the sizes of the bPEI-PL/pDNA NCs were small enough for animal and clinical applications because most available nanoparticles range from 50 to 200 nm. In addition, the zeta potential (approximately 21 mV for bPEI/pDNA NC) slightly increased to 25 mV for bPEI-APL(20%)/pDNA NC but decreased to 16 mV for bPEI-EPL(20%)/pDNA NC (Figure 3B) due to the different degrees of ionization of the two different PL amines (i.e., the primary amines in APL have more positive charges than those in EPL).

### 3.3. Transfection Efficiency and Cytotoxicity of the bPEI-PL/pDNA Nanocomplex

**Figure 4:**
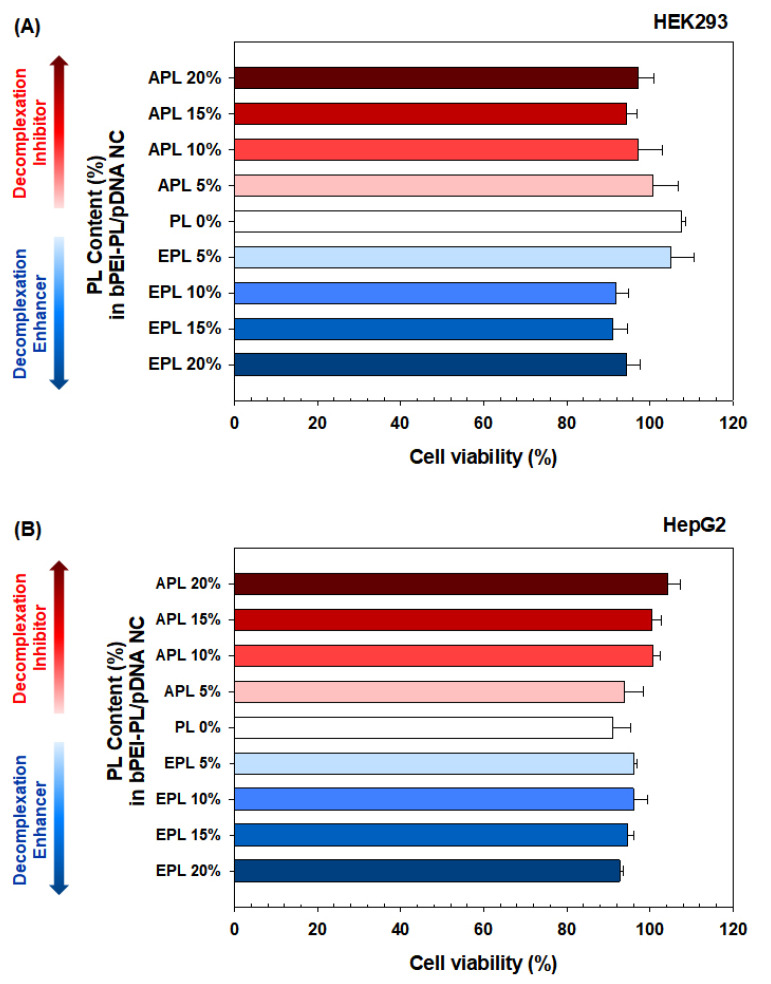
Cell viability of bPEI-PL/pDNA NC-transfected (**A**) HEK293 cells and (**B**) HepG2 cells. Cell viability (%) is expressed as the mean ± standard error (SE) (*n* = 4).

The size and compactness of the bPEI-PL/pDNA NCs could suggest that they possess enough complexation for stable systemic and cellular delivery, and their decomplexation patterns in nucleus-mimicking (i.e., heparin-rich) conditions could allow for faster or slower gene expression rates depending on the PL present. In particular, to exclude the cytotoxicity-mediated effects, the cytotoxicity tests were performed prior to the transfection experiments, and all the tested NCs were found to have negligible cytotoxicity because all the NC-transfected cells had greater than 90% viability (Figure 4). Thus, as a next step, we investigated how a certain balance between complexation and decomplexation (particularly PL-dependent decomplexation rates) of bPEI-PL/pDNA NCs affects transfection efficiency (TE) in an easy-to-transfect cell (HEK293 cells) and a hard-to-transfect cell (HepG2 cells). For easy comparison, the TE of bPEI/pDNA NC was set as unity, and the TEs of the bPEI-PL/pDNA NCs were expressed by normalization to the TE of bPEI/pDNA NC.

**Figure 5:**
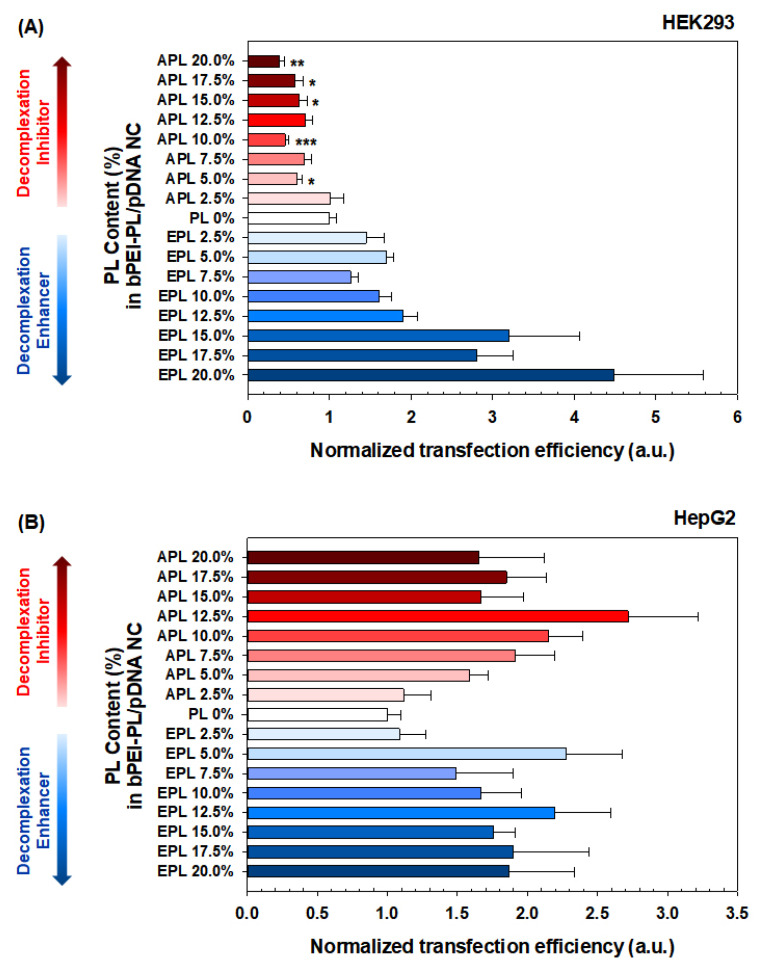
Normalized transfection efficiency (NTE) of bPEI-PL/pDNA NCs in (**A**) HEK293 cells and (**B**) HepG2 cells. NTE is expressed as the mean ± SE (*n* = 8).

When bPEI-PL/pDNA NCs were applied to easy to transfect HEK293 cells, their effects on the TE were obviously affected by either the decomplexation inhibitor (i.e., APL) or decomplexation enhancer (i.e., EPL) (Figure 5A). Namely, with increasing PL content in the NCs, the normalized TEs (NTEs) of bPEI-APL/pDNA NCs gradually decreased from 1.01 (for 2.5% APL) to 0.38 (for 20% APL), whereas the NTEs of bPEI-EPL/pDNA NCs gradually increased from 1.45 (for 2.5% EPL) to 4.48 (for 20% EPL). These results indicate that faster decomplexation of bPEI-based polyplexes, compared with slower decomplexation, could result in higher gene expression in an easy-to-transfect cell.

However, the TE pattern of bPEI-PL/pDNA NCs in a hard-to-transfect HepG2 cell was not similar to that in an easy-to-transfect HEK293 cell (Figure 5B). When applying 2.5% EPL, the NTE of bPEI-EPL(2.5%)/pDNA NCs was 1.08 and similar to that of bPEI/pDNA NC. As expected, increasing EPL contents (5–20%) in bPEI-EPL/pDNA NCs resulted in an average of 1.88-fold higher NTEs than 0% EPL (i.e., bPEI/pDNA NC). However, the dependence of EPL contents on the NTEs of bPEI-EPL(5–20%)/pDNA NCs was not significant. For bPEI-APL(2.5%)/pDNA NC, although APL (2.5%) increased its NTE by approximately 12% compared to the NTE of bPEI/pDNA NC, this difference was not large. Interestingly, with increasing APL content (5–20%), the NTEs of bPEI-APL/pDNA NCs reached a peak at 2.72 (for 12.5% APL) and then decreased to 1.65 (for 20% APL). These results indicate that faster decomplexation of bPEI-PL/pDNA NC than bPEI/pDNA NC in the nucleus could improve the TE in a hard-to-transfect HepG2 cell, similar to the case of the easy-to-transfect HEK293 cell. However, the bell-shaped TE pattern observed for bPEI-APL/pDNA NCs in HepG2 cells might also suggest that other factors affect TE. In particular, the different TE patterns of APL (5–12.5%) between HEK293 cells and HepG2 cells could mean that the certain characteristics of APL (5–12.5%) that improve the TE compensate for the slow decomplexation effects to reduce the TE in HepG2 cells, and that the 15–20% APL characteristics do not have enough influence to improve the TE by overcoming the slow decomplexation-induced decrease in TE.

### 3.4. Proton Buffering Capacities of the Mixtures of bPEI and PL for Estimating Endosomal Escape of Polyplex

**Figure 6:**
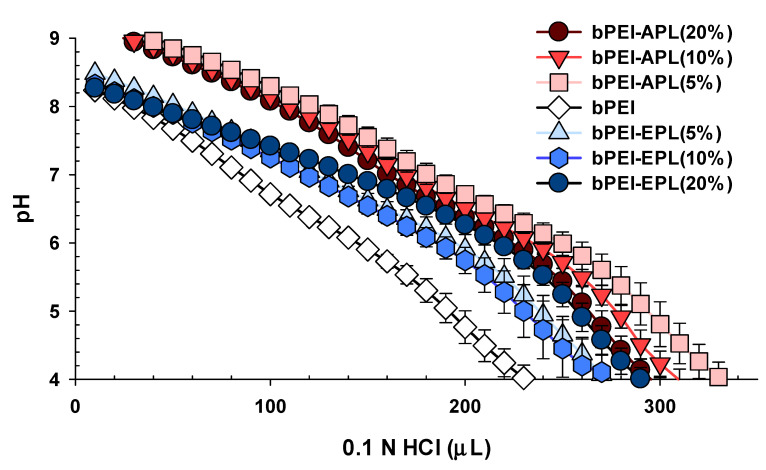
Proton buffering capacities of the mixtures of bPEI and PL in 150 mM NaCl. The data are expressed as the mean ± SD (*n* = 3).

To estimate the endosomal escape of the polyplexes, the proton buffering capacities of the mixtures of bPEI and PL in 150 mM NaCl were tested by acid-base titration. Based on the unit mole-based proton buffering capacity of the mixture in the endosomal pH range of pH 5.1 to 7.4, bPEI exhibited 0.181/μmol with a (+) charge. For the mixtures of bPEI and APL in the bPEI-APL/pDNA NCs, 5%, 10%, and 20% APL corresponded to 0.185, 0.183, and 0.179/μmol with a (+) charge, respectively, whereas the mixtures of bPEI-EPL(5%), bPEI-EPL(10%), and bPEI-EPL(20%) in bPEI-EPL/pDNA NCs corresponded to 0.195, 0.195, and 0.219/μmol with a (+) charge, respectively (Figure 6). Namely, the proton buffering activity levels of the bPEI-APL mixtures were similar to those of bPEI, whereas the bPEI-EPL mixtures had higher proton buffering activity levels than bPEI. These results reflected our previous results showing that EPL had 3.4-fold and 1.9-fold higher proton buffering capacity than APL and bPEI, respectively [24], and suggested that the bPEI-EPL/pDNA NCs could escape from endolysosomes earlier than the bPEI/pDNA NCs or bPEI-APL/pDNA NCs.

### 3.5. Cellular Uptake, Nuclear Uptake, and Subcellular Localization of the bPEI-PL/pDNA Nanocomplex

**Figure 7:**
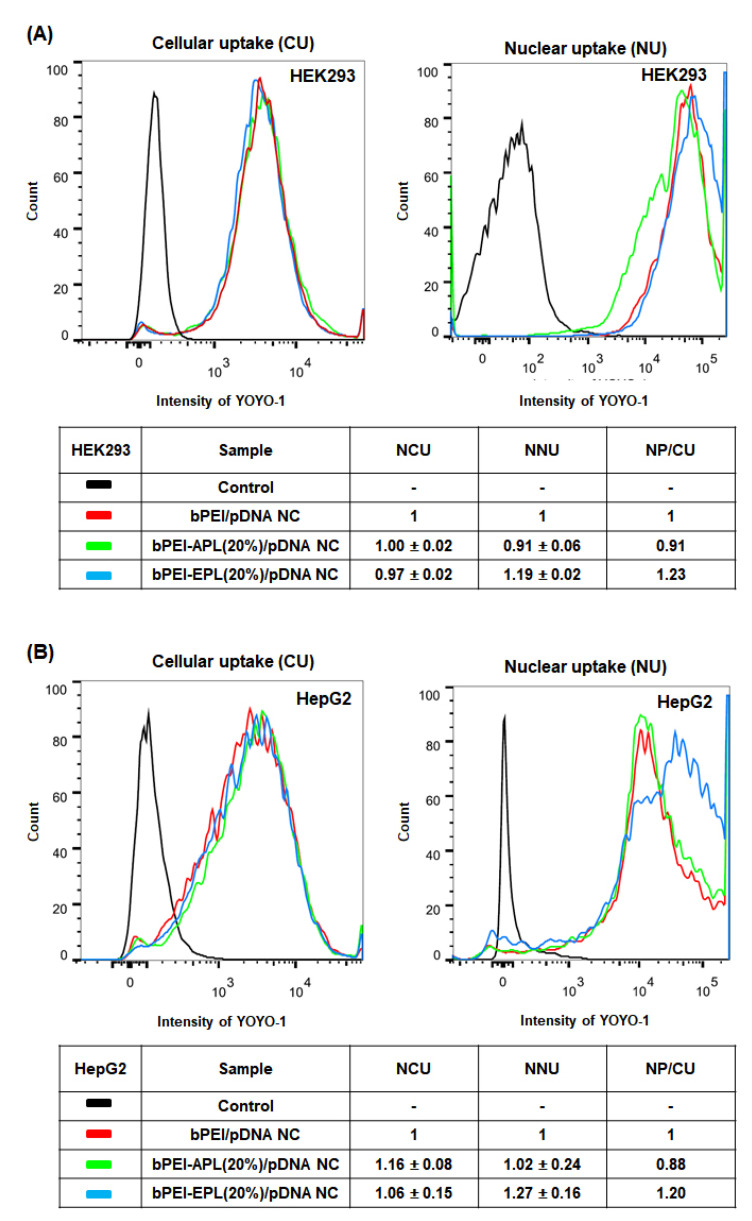
Cellular uptake (CU) and nuclear uptake (NU) of bPEI-PL/pDNA NCs in (**A**) HEK293 cells and (**B**) HepG2 cells. Normalized CU (NCU) and normalized NU (NNU) are expressed as the mean ± SE (*n* = 3), and NP/CU was expressed as the mean (*n* = 3).

To understand the different TE effects of bPEI-PL/pDNA NCs in the two different cell lines, their cellular uptake (CU), nuclear uptake (NU), and subcellular localization were monitored because these characteristics, along with decomplexation, strongly affect their TE. In particular, bPEI-APL (20%)/pDNA NC and bPEI-EPL (20%)/pDNA NC were used as the model NCs of bPEI-PL/pDNA NCs, and are simply called as APL (20%) and EPL (20%), respectively. In HEK293 cells, APL (20%) exhibited a similar CU to the control (i.e., bPEI/pDNA NC), whereas the CU of EPL (20%) was approximately 3% lower than that of the control and APL (20%) (Figure 7A). The results might be caused by different physicochemical characteristics (e.g., size, zeta potentials) of bPEI-PL/pDNA NCs because EPL (20%) (123 nm, 16.1 mV) had a relatively larger size and lower zeta potential than those of APL (20%) (63 nm, 25.2 mV) and the control (78 nm, 21.1 mV). However, the NU of APL (20%) was 0.91-fold and 0.76-fold lower than those of the control and EPL (20%), respectively (Figure 7A). Interestingly, although EPL (20%) had a similar CU as that of APL (20%) (0.97 versus 1.00), the former represented 1.31-fold higher NU than the latter. For easier comparison, when the NU is divided by the CU, the nuclear preference to CU (NP/CU) of EPL (20%) was 1.35-fold higher than that of APL (20%) (1.23 versus 0.91). These results could be influenced by the different endosomolytic activities of the two PLs because EPL has a proton buffering-mediated endosomal escape ability, unlike APL (Figure 6), and both APL and EPL have nuclear translocating activities after endosomal escape [7,24]. The additional endosomal escape activity of EPL could help EPL (20%) escape from endolysosomal compartments earlier than the control, whereas the lack of proton buffering power from APL could cause APL (20%) escape from endolysosomal compartments later than the control.

In HepG2 cells, although both APL (20%) and EPL (20%) had a greater CU than the control, the CU of APL (20%) was slightly (1.09-fold) larger than that of EPL (20%) (1.16 versus 1.06) (Figure 7B). Additionally, the NU of EPL (20%) was 1.25-fold greater than that of APL (20%) (Figure 7B). As a result, the former’s NP/CU was 1.36-fold higher than the latter’s NP/CU (1.20 versus 0.88). The CU, NU, and NP/CU results in that HepG2 cells were similar to those in HEK293 cells. These facts suggest that the effects of the particular PL on the CU, NU, and NP/CU of bPEI-PL/pDNA NCs should not be influenced by the transfection difficulty of the cell. Additionally, it could be inferred that the decomplexation of the PL from the bPEI-PL/pDNA NCs mostly occurred in the nucleus, but also in other subcellular organelles.

**Figure 8:**
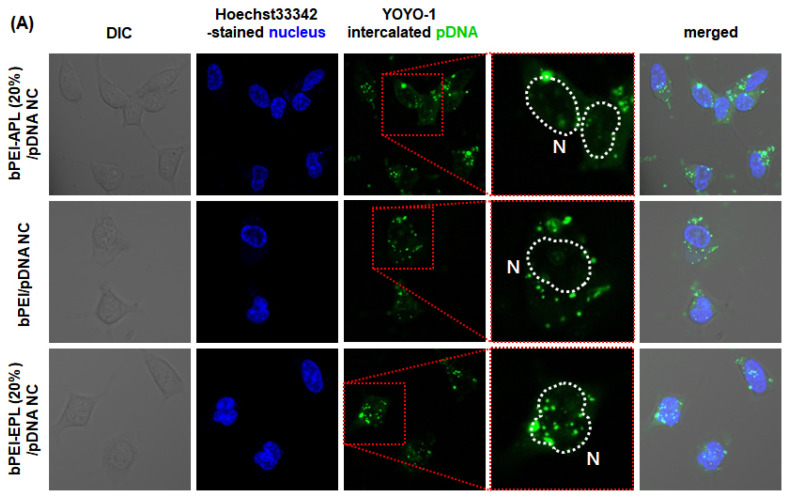

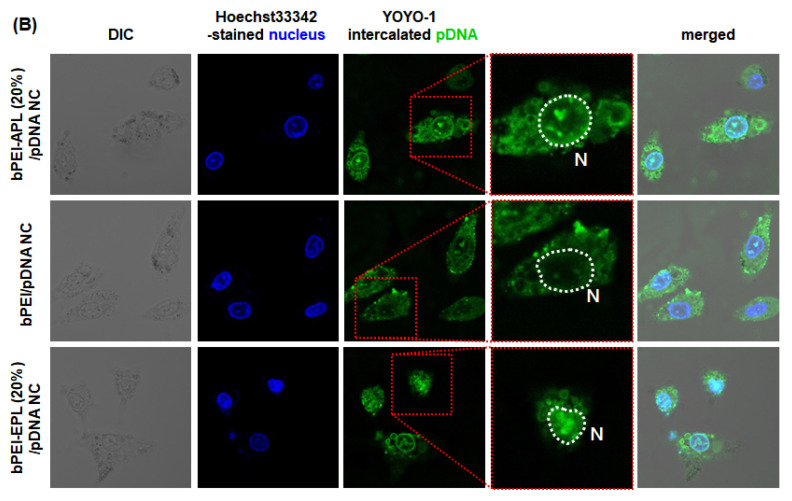
Intracellular distribution of YOYO-1-intercalated pDNA delivered by bPEI-PL/pDNA NC in (**A**) HEK293 cells and (**B**) HepG2 cells 4 h post-transfection.

**Figure 9:**
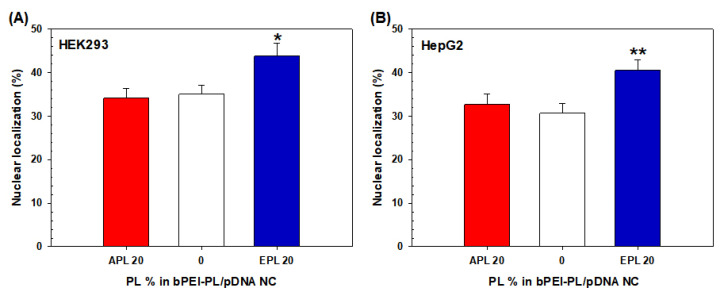
Nuclear localization of bPEI-PL/pDNA NC in (**A**) HEK293 cells and (**B**) HepG2 cells. The data are expressed as the mean ± SE (*n* > 20). (* *p* < 0.05, ** *p* < 0.01 compared with bPEI/pDNA NC).

To support the NP/CU results of bPEI-PL/pDNA NCs calculated by flow cytometry (Figure 7), its nuclear localization was further visualized and estimated by confocal microscopy (Figure 8 and Figure 9). As shown in Figure 8A,B, bPEI/pDNA NC was mostly present in the cytosol, but the NC was rarely found in the nucleus. Compared to bPEI/pDNA NC, more APL (20%) and EPL (20%) were localized in the nucleus. In particular, EPL (20%) was distributed in the nucleus to a greater extent than APL (20%). In addition, the calculated nuclear localization percentages of APL (20%) and EPL (20%) in HEK293 cells and HepG2 cells were 34.2% and 32.7%, respectively, for APL (20%) and 43.8% and 40.5%, respectively, for EPL (20%) (Figure 9A,B). The nuclear localization values of EPL (20%) were 1.28-fold (in HEK293 cells) and 1.24-fold (in HepG2 cells) higher than those of APL (20%). These nuclear localization confocal results of the APL (20%) and EPL (20%) agree with their flow cytometry results. The results indicate that the nuclear localization trends of bPEI-PL/pDNA NCs are not affected by the transfection difficulty of the cells.

Interestingly, although the amount of pDNA delivered with bPEI-PL/pDNA NCs was same, more pDNA was localized in the nucleus of HepG2 cells than that of HEK293 cells (Figure 8), their TEs in HEK293 cells were much higher than those in HepG2 cells (Figure 5). These results could be explained by the fact that transfection efficiency is highly dependent on transcription and translation [33]. In particular, HEK293 cells exhibit high transfection efficiency because of the faithful translation and processing of proteins [34]. Consistent with these facts, many reports have shown that HEK293 cells have high transfection efficiency but that HepG2 cells have low transfection efficiency [27,35]. Thus, although the amount of pDNA in the nucleus strongly correlates with the gene expression level in the cells of the same lineage, the same amount of pDNA in the nucleus of different cell lines may express different levels of proteins. In particular, a smaller amount of pDNA in the nucleus of the HEK293 cells compared with that in the nucleus of HepG2 cells may indicate a higher transfection efficiency for the HEK293 cells than that for the HepG2 cells because the HEK293 and HepG2 cells are an easy-to-transfect cell and a hard-to-transfect cell, respectively.

**Figure 10:**
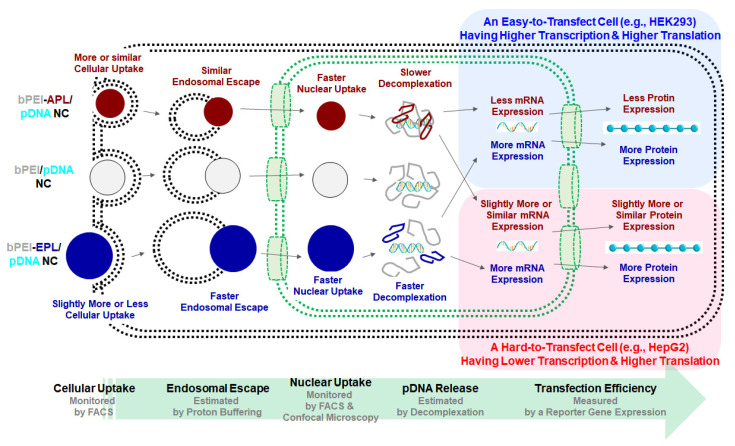
The effects of PL on the intracellular transfection steps of the bPEI-PL/pDNA NCs in either an easy-to-transfect cell or a hard-to-transfect cell.

Overall, this study attempted to control the decomplexation rates of bPEI-based gene complexes in the nucleus using either a decomplexation inhibitor (APL) or a decomplexation enhancer (EPL) because APL and EPL cause strong and weak gene-holding forces, respectively [7,24]. APL-induced strong gene complexation made the bPEI-APL/pDNA NCs smaller than the bPEI/pDNA NCs, whereas EPL-induced weak gene complexation caused the bPEI-EPL/pDNA NCs to be larger than the bPEI/pDNA NCs (Figure 3). Nevertheless, both bPEI-APL/pDNA NCs and bPEI-EPL/pDNA NCs exhibited similar pDNA compactness (Figure 2). As expected, in a heparin-induced decomplexation study, bPEI-APL/pDNA NCs showed slower decomplexation than bPEI/pDNA NC, whereas bPEI-EPL/pDNA NCs represented faster decomplexation than bPEI/pDNA NC (Figure 1). When applying the decomplexation-enhancing bPEI-EPL/pDNA NCs to an easy-to-transfect HEK293 cell or a hard-to-transfect HepG2 cell, EPL strongly improved the TE of bPEI-based NCs regardless of the transfection difficulty of the cells (Figure 5). However, when transfecting bPEI-APL/pDNA NCs into these two cells, their effects on the TE were different: a decomplexation inhibitor-induced TE drop was observed in HEK293 cells and a bell-shaped TE pattern was obtained in HepG2 cells (Figure 5). In particular, the TE of a slow decomplexation system in a hard-to-transfect cell could be affected by the decomplexation rate as well as other factors, because the effects of the PL on the CU, NU, and NP/CU were not affected by the transfection difficulty of the cell (Figure 7, Figure 8, Figure 9). Of course, although the bPEI-PL/pDNA NCs exhibited different endosomal escape activity levels, depending on the incorporation of APL or EPL in the NCs (Figure 6), the NU and NP/CU of the NCs reflected the differences in their endosomal escape. Therefore, as with other factors, PL-mediated enhanced nuclear localization of the bPEI-PL/pDNA NCs could be considered. Introducing a PL into bPEI-based gene complexes increased the nuclear delivery of NCs and simultaneously tuned their decomplexation rates in the nucleus. When applying EPL as the PL, both the higher NP/CU and faster decomplexation rate compared with bPEI/pDNA NCs enhanced transfection regardless of the transfection difficulty of the cell. However, although APL could help bPEI-APL/pDNA NCs have a higher NP/CU than that of the bPEI/pDNA NCs, APL could limit the decomplexation of bPEI-APL/pDNA NCs in the nucleus. Namely, a reducing factor (i.e., slow decomplexation) in the TE will be compensated for by an improving factor (i.e., improved nuclear delivery) in the TE. In an easy-to-transfect cell, a slow decomplexation could be a major contributor to determining the TE compared to a high NP/CU because the cell is generally proliferating at a high rate. However, in a hard-to-transfect cell, which is generally growing at a slow rate, a high NP/CU could be a major contributor to gene expression compared to slow decomplexation. Thus, a decomplexation controller could be used to tune and increase or decrease the TE. In particular, although the TE in an easy-to-transfect cell could be strongly affected by the decomplexation rate, the TE in a hard-to-transfect cell could result from compensation from multiple factors such as a decomplexation rate and a high NP/CU. The overall effects of PL on the intracellular transfection steps of the bPEI-PL/pDNA NCs in either an easy-to-transfect cell or a hard-to-transfect cell are graphically summarized in the Figure 10.

## 4. Conclusions

In this study, two different decomplexation controllers were incorporated into bPEI-based gene complexes to understand how the decomplexation rates of gene complexes in the nucleus affect the TE in different cells with different transfection difficulties. As expected, a decomplexation enhancer (EPL) improved the TE of gene complexes with improved nuclear uptake regardless of the transfection difficulty of the cell. However, although a decomplexation inhibitor (APL) could reduce the TE of gene complexes regardless of the transfection difficulty of gene complexes, the reduced TE could be decreased, neutralized, or overcome by the improved nuclear uptake-mediated compensation depending on the use of either a hard-to-transfect cell or an easy-to-transfect cell. As a result, the decomplexation rate in the nucleus could be a potential factor to design effective gene delivery systems with high transfection efficiency.

## Supplementary Materials

The following are available online at https://www.mdpi.com/1999-4923/12/6/490/s1, Figure S1: Size distributions of bPEI-PL/pDNA NCs, Figure S2: TEM images of bPEI-PL/pDNA NCs.
