# Supplementary material for: Effects of Decomplexation Rates on Ternary Gene Complex Transfection with α-Poly(l-Lysine) or ε-Poly(l-Lysine) as a Decomplexation Controller in An Easy-To-Transfect Cell or A Hard-To-Transfect Cell
Source: Pharmaceutics. 2020 May 28;12(6):490. doi: 10.3390/pharmaceutics12060490 (PMC7356167; doi:10.3390/pharmaceutics12060490)
Supplement: Supplementary file 1 [file pharmaceutics-12-00490-s001.pdf]

pharmaceutics

MDPI

Supplementary Materials: Effects of Decomplexation Rates on Ternary Gene Complex Transfection with α-Poly(L-Lysine) or ε-Poly(L-Lysine) as a Decomplexation Controller in An Easy-To-Transfect Cell or A Hard-To-Transfect Cell

Kyoungnam Kim, Kitae Ryu, Hana Cho, Min Suk Shim, Yong-Yeon Cho,
Joo Young Lee, Hye Suk Lee and Han Chang Kang

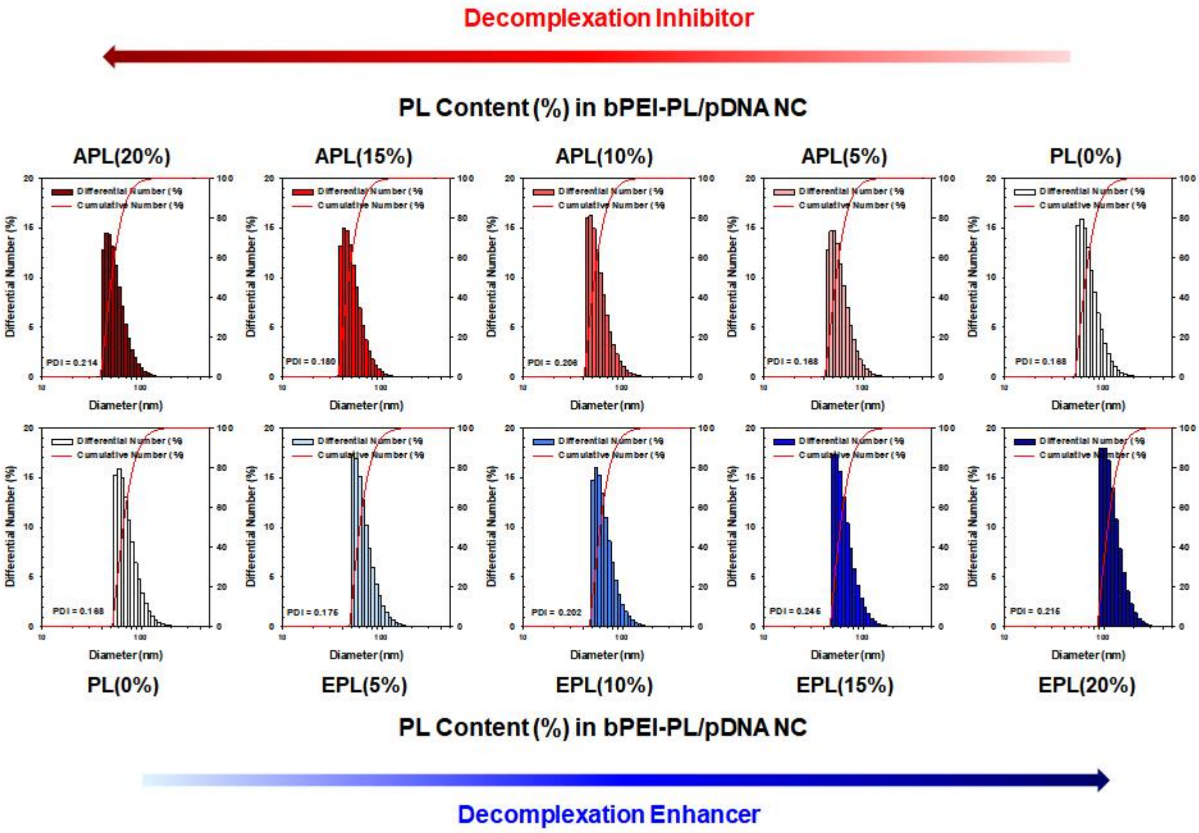

Figure S1. Size distributions of bPEI-PL/pDNA NCs.

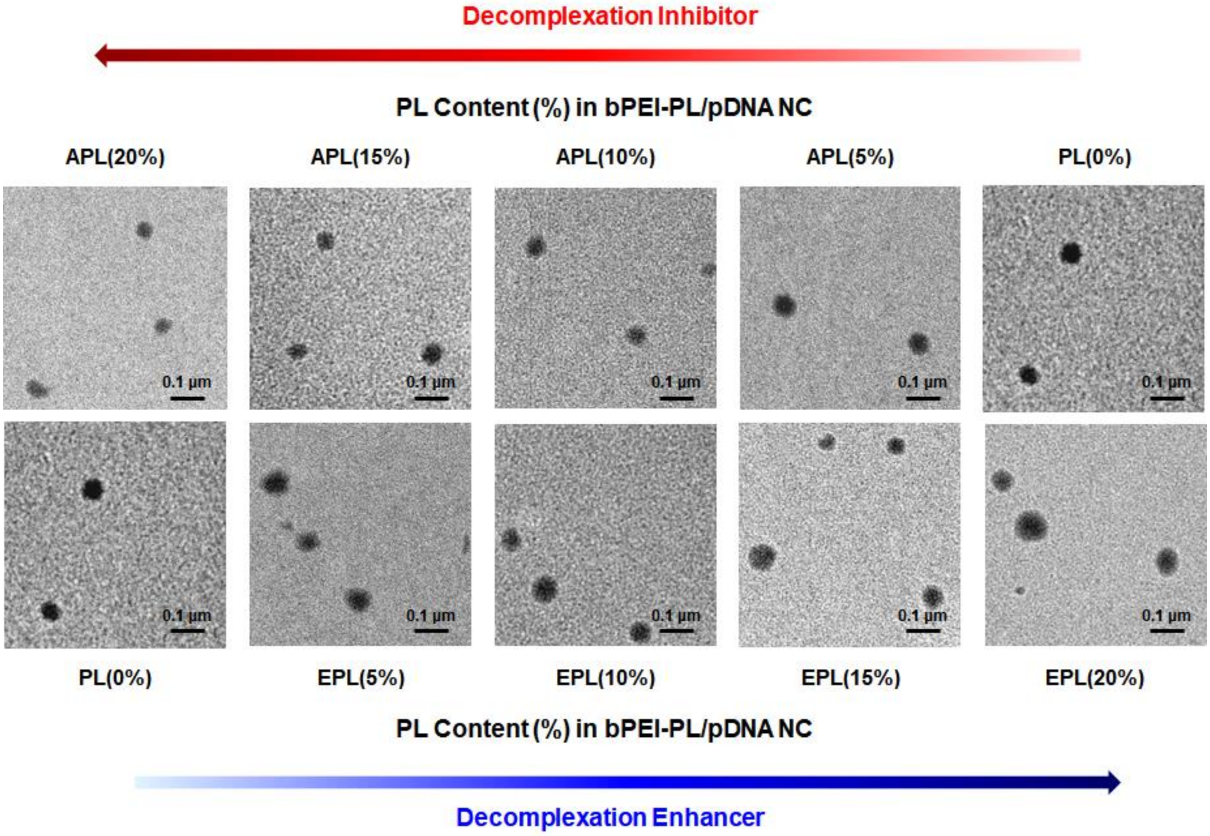

Figure S2. TEM images of bPEI-PL/pDNA NCs (scale bar: 0.1 µm).
